# Hybrid Deep–Geometric Approach for Efficient Consistency Assessment of Stereo Images

**DOI:** 10.3390/s25144507

**Published:** 2025-07-20

**Authors:** Michał Kowalczyk, Piotr Napieralski, Dominik Szajerman

**Affiliations:** Institute of Information Technology, Lodz University of Technology, al. Politechniki 8, 93-590 Lodz, Polandpiotr.napieralski@p.lodz.pl (P.N.)

**Keywords:** stereoscopic images, geometric consistency, transformer-based object detection, semantic inconsistency, epipolar geometry, stereo vision, Middlebury benchmark, feature matching

## Abstract

**Highlights:**

**What are the main findings?**

- We propose a self-contained, single-pair stereo consistency check that fuses epipolar geometry with Transformer-based object detection.
- Our method flags both global camera misalignments and localized semantic or geometric anomalies without external calibration data.

**What is the implication of the main finding?**

- Enables on-the-fly quality assurance of stereo rigs in applications from robotics to 3D cinematography.
- Lays groundwork for combining semantic scene understanding with classical stereo geometry.

**Abstract:**

We present HGC-Net, a hybrid pipeline for assessing geometric consistency between stereo image pairs. Our method integrates classical epipolar geometry with deep learning components to compute an interpretable scalar score A, reflecting the degree of alignment. Unlike traditional techniques, which may overlook subtle miscalibrations, HGC-Net reliably detects both severe and mild geometric distortions, such as sub-degree tilts and pixel-level shifts. We evaluate the method on the Middlebury 2014 stereo dataset, using synthetically distorted variants to simulate misalignments. Experimental results show that our score degrades smoothly with increasing geometric error and achieves high detection rates even at minimal distortion levels, outperforming baseline approaches based on disparity or calibration checks. The method operates in real time (12.5 fps on 1080p input) and does not require access to internal camera parameters, making it suitable for embedded stereo systems and quality monitoring in robotic and AR/VR applications. The approach also supports explainability via confidence maps and anomaly heatmaps, aiding human operators in identifying problematic regions.

## 1. Introduction

Stereoscopic imaging enables depth perception by capturing a scene from two slightly different viewpoints, mimicking human binocular vision. It has been widely adopted across domains—from medicine and robotics to autonomous vehicles and entertainment—to facilitate reasoning about 3D structure [1]. However, the accuracy of any stereo vision system critically depends on the geometric consistency between the two views. Even minor misalignments or calibration errors between stereo cameras can lead to geometric distortions that undermine depth estimation algorithms and degrade visual quality [2]. Misaligned stereoscopic content may cause incorrect 3D measurements and, in human-viewed systems like VR/AR, can induce eye strain or motion sickness [3]. Ensuring that the two images of a stereo pair are geometrically consistent (i.e., conforming to the assumed epipolar geometry) is therefore essential for both machine and human interpreters of stereo data [4].

Geometric consistency in this context means that the relative camera geometry (orientation, focal alignment, etc.) is consistent with the stereo image pair, so that corresponding points in the scene lie on the same epipolar line in both images. Traditional solutions to enforce or evaluate this consistency have relied on explicit camera calibration procedures (e.g., using known calibration patterns or multiple images to estimate intrinsic/extrinsic parameters). While effective, such methods are labor-intensive and not always feasible in the field, where the calibration may drift over time or where only a single stereoscopic image pair is available. There is a clear need for automatic methods to assess stereo image geometric alignment from the image data alone, without requiring special calibration targets or prior camera parameters [5]. In stereo vision, ensuring geometric consistency typically involves verifying that corresponding points lie along matching epipolar lines—a constraint rooted in the epipolar geometry induced by the stereo camera setup.

Recently, Kowalczyk [6] proposed an efficient method for geometric consistency evaluation of stereoscopic images that operates on a single stereo pair without any calibration input. This method leverages epipolar geometry analysis: by finding correspondences between the left and right image and analyzing the epipolar lines, it can quantify how well the stereo pair adheres to an ideal pinhole stereo model. The approach is deterministic, automatic, and requires minimal data – only the stereo images themselves—yet achieves very high estimation accuracy. In essence, it provides a quantitative geometric consistency score from 0 to 1 indicating the degree of alignment (with 1 meaning a perfectly consistent stereo geometry). This original method forms the backbone of our work. However, the approach by Napieralski and Kowalczyk [6] has certain limitations: it relies purely on classical feature matching (which can fail in low-texture or degenerate scenarios) and assumes an ideal pinhole camera model with negligible distortion. Moreover, because it uses no learned scene information, it cannot flag semantic or content anomalies in the stereo pair. These shortcomings motivate the enhancements proposed in this paper.

**Figure 1:**
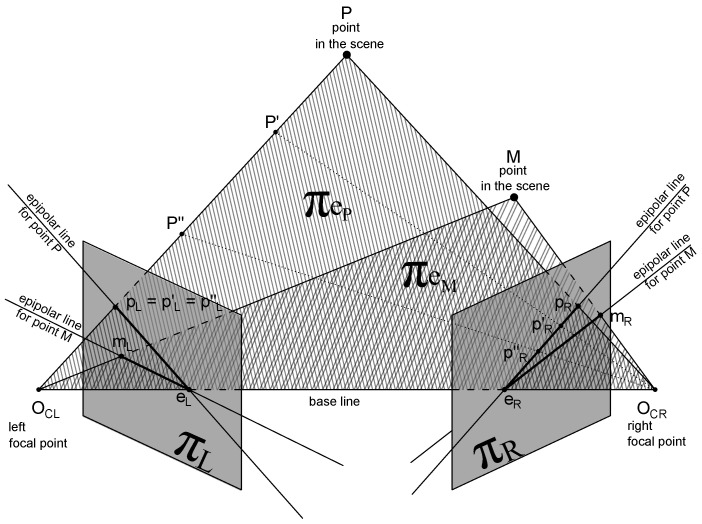
Epipolar geometry of a stereo acquisition system: each 3D point *P* projects to a pair of image points $p_{L}$ and $p_{R}$ lying on corresponding epipolar lines $l_{L}$ and $l_{R}$.

To address these limitations, we propose a fully deterministic and self-contained method for evaluating geometric consistency directly from a single stereo pair. The method estimates the fundamental matrix using robust feature matching and outlier rejection, and derives consistency indicators based on the deviation of epipolar line parameters. This enables accurate, calibration-free evaluation with minimal computational cost, making it particularly suitable for embedded or real-time systems. As illustrated in Figure 1, the approach models each 3D point as projecting to image coordinates that must lie on corresponding epipolar lines; deviations in slope or offset signal geometric distortion.

At the same time, the past five years have seen rapid advances in deep learning for stereo vision. Tasks such as stereo matching and depth estimation have greatly benefited from convolutional neural networks (CNNs) and, more recently, Transformer-based architectures [7]. Researchers have also begun applying learning-based methods to stereo geometry problems—for example, using CNNs to estimate the fundamental matrix or to judge whether a stereo pair is rectified (canonical). Notably, Poursaeed et al. [8] introduced a deep network that directly estimates the fundamental matrix from an image pair without needing explicit feature correspondences. Other work trained CNNs to detect if stereo images are in proper canonical form (i.e., with horizontal epipolar lines). Vision Transformers have been used to establish dense correspondences across images (e.g., LoFTR uses self-attention to produce high-quality matches even in low-texture regions [9], and 3D CNNs operating on cost volumes have improved disparity computation. These developments suggest that incorporating learned features or networks into the geometry consistency assessment could further enhance its robustness and accuracy. In this paper, we unite the classical geometric approach with modern deep learning techniques to create a hybrid stereoscopic image consistency evaluation method. We present a clear exposition of an epipolar geometry-based consistency evaluation algorithm that requires no calibration data. We describe its mathematical model and demonstrate its efficiency and accuracy on single stereo pairs. We extend the above method by incorporating deep learning techniques, including Transformer-based correspondence networks and CNN-based feature matchers, creating a hybrid approach that leverages both geometric constraints and learned representations. To our knowledge, this is one of the first works to merge classical epipolar analysis with modern deep models for this task.

The main contributions of this work can be summarized:

- We propose a hybrid geometric consistency assessment method (HGC-Net) that fuses classical epipolar geometry analysis with deep learning techniques.
- The core metric, a scalar score *A*, quantifies stereo alignment from a single image pair without requiring calibration or depth ground truth.
- We enhance robustness and accuracy by integrating deep feature matchers (e.g., LoFTR), Transformer-based disparity estimators (e.g., STTR), and attention-based diagnostic modules.
- Our method outperforms baseline techniques in detecting both global miscalibration and local geometric anomalies, achieving correlation with simulated distortion levels.

To the best of our knowledge, this is the first method to combine no-reference geometric evaluation with learned components in a unified, interpretable framework.

## 2. Related Work

### 2.1. Stereo Geometric Consistency and Calibration Methods

A longstanding approach to ensure stereo image consistency is rigorous camera calibration. Traditional calibration techniques (e.g., using the method of Zhang) estimate intrinsic and extrinsic parameters of each camera by observing a known target (checkerboard, etc.), after which images can be rectified to align epipolar lines [10]. Any geometric inconsistencies can then be detected by monitoring changes in these parameters or measuring reprojection errors. In practice, however, stereo rigs may become miscalibrated due to mechanical shifts (vibrations, temperature, etc.), and performing a full calibration in the field is impractical. Researchers have thus explored automatic calibration or self-calibration methods that rely on feature correspondences in the scene. For example, one can match feature points between the left and right images, compute the fundamental matrix F using the normalized eight-point algorithm, and then compare F to the ideal configuration (or decompose F to check for known baseline geometry). The fundamental matrix encapsulates the cameras’ relative orientation; inconsistencies manifest as deviations in F’s structure (e.g., epipoles not at infinity when they should be, etc.). Classic metrics include the mean epipolar alignment error—e.g., the average distance of matched points to the epipolar lines of their counterparts—or the distribution of vertical disparities after rectification. Some recent works have studied how specific angular errors affect stereo geometry; for example, Felipe et al. [11] analyzed the impact of pitch angle deviations on calibration outcomes and proposed a correction scheme based on optimization. While their method improves robustness under angular misalignment, it still assumes access to approximate baseline geometry and requires model-driven adjustment. These calibration-based evaluations require robust matching and outlier removal, but can achieve good accuracy when many points are available.

### 2.2. Disparity-Based Consistency Metrics

Another category of methods assess stereo consistency via the disparity map (depth map) obtained from the stereo pair [12]. If the images are well-aligned, standard stereo matching algorithms (block matching, SGM, etc.) will produce a coherent disparity map with minimal artifacts. Conversely, geometric distortions (like vertical parallax or camera tilt) typically yield disparity computations with systemic errors. One simple consistency check is the left–right disparity check: compute disparities from left to right and right to left and measure their differences—large inconsistencies indicate misalignment. Prior works have used the percentage of bad disparity pixels or disparity error statistics as an indicator of stereo geometry problems. For instance, an algorithm might compute a full-resolution disparity using a semi-global matcher and then evaluate how many disparities violate epipolar constraints or differ when swapping left/right roles. While this can flag severe miscalibration, it is computationally costly (disparity estimation itself is intensive) and can be confounded by general image quality issues (textureless regions, etc.). In addition, disparity-based metrics often require a reference for evaluation or assume the majority of the scene is static and visible to both cameras, which might not hold in all cases.

### 2.3. No-Reference Stereoscopic Image Quality (NR-SIQA)

More broadly, the field of stereoscopic image quality assessment addresses how to predict the perceptual quality of a stereo pair without a reference image. Recent NR-SIQA methods use machine learning to handle various distortion types, including geometric misalignment. For example, Li et al. [13] developed algorithms that extract statistical features or deep features from the stereoscopic images (sometimes via a synthesized “cyclopean” view) to predict a quality score. Deep learning has made significant inroads here: one 2021 approach uses a four-channel deep network taking the left image, right image, and binocular difference to predict quality [14]. More recently, an end-to-end NR-SIQA neural network was proposed that generates patches guided by a saliency map and feeds them into a deep model to output a quality prediction [15]. These works primarily focus on perceptual quality (sharpness, compression artifacts, etc.), but implicitly, a well-aligned stereo pair will score higher than one with misalignment, since human quality opinions are affected by view inconsistency. However, such methods do not explicitly quantify geometric consistency nor explain the nature of the distortion. In contrast, our work aims to directly measure geometric alignment quality in a physically interpretable way (related to camera geometry parameters), though we can certainly leverage similar deep features to aid the assessment.

### 2.4. Learning-Based Approaches for Stereo Geometry

In the last five years, researchers have begun applying deep learning to estimate geometric alignment and calibration parameters from images. One notable example is the work of Poursaeed et al., who introduced deep networks to estimate the fundamental matrix F directly from raw image pairs [8]. By formulating the problem in an end-to-end manner and introducing layers that enforce the rank-2 constraint of F, their approach can handle scenarios with large viewpoint changes or occlusions where traditional feature-based F estimation might fail. The network essentially learns to correlate patterns between the two images and output the 7-degree-of-freedom fundamental matrix. Another line of research uses CNNs for stereo rectification verification: e.g., Morra et al. [16] trained a convolutional model to automatically detect if a stereo image pair is in canonical orientation (aligned horizontally) or if it requires rectification. Such a classifier can serve as a preliminary check for gross misalignment (for instance, detecting rotations or vertical offsets that break canonicity). Graph-based neural networks and attention mechanisms have also been leveraged for stereo correspondence, which relates to geometry consistency. SuperGlue [9] is a graph neural network that takes detected feature points from two images and learns to match them, significantly outperfoming heuristic matching; this can provide more reliable correspondences for downstream geometry estimation. Similarly, Transformer-based matchers like LoFTR (Local Feature Transformer) remove the need for explicit detection and instead find a dense set of matches using self- and cross-attention over the image pair. LoFTR is robust to low-texture and repetitive patterns, yielding high-quality correspondences even where traditional methods struggle. These learned matching techniques are directly relevant to our problem: by plugging them into the classical pipeline (in place of, say, ORB or SIFT feature matching), we can improve the resilience of geometric estimation under difficult imaging conditions. We will later demonstrate how integrating a deep matcher increases the reliability of our epipolar geometry analysis, especially in challenging scenes. Stereo Matching and Depth Estimation Networks: Although depth estimation per se is not our end goal, modern stereo matching networks provide useful insights and tools. Many state-of-the-art stereo depth networks use a 3D convolutional neural network (3D CNN) on a cost volume (a tensor encoding matching costs for each pixel at various disparity candidates) to infer disparities. For example, PSMNet and GANet (2018–2019) achieved top performance using stacked hourglass 3D CNNs to aggregate matching information. More recently, Transformer-based models have emerged: STTR (Stereo Transformer) by Li et al. reformulates stereo matching as a sequence-to-sequence prediction using attention, eliminating the need for a fixed disparity range [7]. STTR can naturally handle occlusions (by identifying unmatched regions) and produces a confidence map for disparities. The trend is to incorporate geometric priors (like epipolar constraints) into deep networks while exploiting their ability to learn from data [17]. These advanced stereo networks implicitly learn when a pair of images is inconsistently aligned—often, they will output erroneous or uncertain disparities if the input violates the assumed geometry. In a sense, a robust depth network could be used as a consistency oracle: if it fails to find a coherent disparity map, the geometry is likely off. Some works have proposed training deep models to output a binary decision on stereo consistency or to refine calibration. For instance, Yang et al. [18] used canonical correlation analysis networks to improve two-view alignment for recognition tasks. Multi-View and Anomaly Detection: Beyond stereo, multi-view geometry consistency is enforced in structure-from-motion and SLAM systems. Those systems use bundle adjustment to maintain a consistent pose estimation across many images. While not directly our focus, they share the goal of detecting geometry outliers. Recently, anomaly detection using multi-view or stereo input has gained attention in specific applications. For example, RADIUS [19] is a framework for anomaly inspection in sewer pipes using stereovision, where a 3D point cloud of the pipe interior is reconstructed from stereo and analyzed for irregularities like cracks or deformations. This demonstrates stereo consistency used in a practical context: the stereo rig must be geometrically reliable to produce accurate 3D reconstructions of the pipe; otherwise, false anomalies could be reported. In autonomous driving, stereo cameras are often part of the sensor suite, and ensuring their calibration is vital—some works have looked at continuous self-calibration for stereo rigs in vehicles (e.g., by optimizing feature tracks over time) [20]. Many prior studies have tackled aspects of stereo image quality and geometry, our work sits at their intersection: providing a dedicated solution to evaluate geometric fidelity of stereo pairs, and doing so by harnessing the best of both model-based and learning-based approaches. In the next section, we detail the methodology of our proposed system, starting from the base classical method and then describing how we integrate deep learning components to address its limitations and improve performance.

## 3. Materials and Methods

### 3.1. Classical Epipolar Geometry Method (Base Algorithm)

For a given stereo pair (left image and right image), if the cameras have a consistent geometric relationship, all corresponding points lie on pairs of conjugate epipolar lines—i.e., the projection of a 3D point will appear at some position in the left image and at a position in the right image constrained to a specific line (the epipolar line) determined by the camera poses. In an ideal rectified stereo setup, these epipolar lines are horizontal scanlines of the images. Geometric inconsistencies (from miscalibration) manifest as tilted or vertically shifted epipolar lines between the two images. The core idea of the base method is to quantitatively measure such deviations.

The algorithm begins by finding a sparse set of feature correspondences between the left and right images. Keypoints are detected in both images, and feature descriptors are extracted (for example, using ORB or SURF features). The descriptors from the two images are then matched to identify candidate corresponding points.

To increase reliability, standard outlier rejection techniques are applied:

- Lowe’s ratio test—to ensure unique best matches;
- symmetry check—keeping only matches that are mutual;
- robust estimation via RANSAC—to filter any remaining incorrect matches.

**Figure 2:**
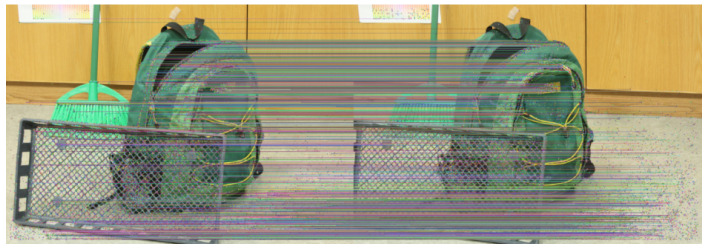
Matched feature points between stereo images after outlier rejection. The horizontal alignment of lines indicates good geometric consistency.

By the end of this process, we obtain a set of *n* putative corresponding point pairs $\left(p_{Li},p_{Ri}\right)$ that represent the same 3D scene points in the left (L) and right (R) images. Figure 2 illustrates an example of matched features after outlier filtering, yielding mostly horizontal connection lines between images.

Using these correspondences, the fundamental matrix *F* is computed. *F* is a 3×3 matrix encoding the epipolar geometry; we use the normalized eight-point algorithm to solve for *F*, ensuring that

(1) $$p_{Ri}^{T}Fp_{Li}=0,$$

for all inlier correspondences. The fundamental matrix *F* relates any point in the left image to an epipolar line in the right image, and $F^{T}$ does the same for a point in the right image to a line in the left image. Once *F* is known, we can derive the equations of epipolar lines in each image for each correspondence.

For each matched point $p_{Li}$ in the left image, we compute its corresponding epipolar line in the right image as

(2) $$\ell_{Ri}=F\left[\begin{array}{c} p_{Li}\\ 1 \end{array}\right],$$

and similarly,

(3) $$\ell_{Li}=F^{T}\left[\begin{array}{c} p_{Ri}\\ 1 \end{array}\right]$$

is the epipolar line in the left image for the right point. We then express each line in slope–intercept form:

(4) $$\ell_{Li}:y=a_{i}^{L}x+b_{i}^{L},\;\ell_{Ri}:y=a_{i}^{R}x+b_{i}^{R},$$

where $a_{i}^{L}$ is the slope and $b_{i}^{L}$ is the vertical intercept of the *i*-th epipolar line in the left image (similarly $a_{i}^{R}$, $b_{i}^{R}$ for the right image).

If the stereo geometry is perfect, these lines should be horizontal and aligned between views: ideally, $a_{i}^{L}=0$ and $a_{i}^{R}=0$ (horizontal lines), and $b_{i}^{L}=b_{i}^{R}$ (aligned in vertical position). Systematic deviations from these ideal values indicate geometric distortion, and more specifically, the following:

- Non-zero slope ($a_{i}^{L}$ or $a_{i}^{R}\neq 0$) means the epipolar line is tilted, suggesting the cameras are rotated relative to the horizontal baseline (e.g., pitch or roll).
- A difference in intercept ($b_{i}^{L}\neq b_{i}^{R}$) indicates vertical offset between left and right epipolar lines, often caused by camera height mismatch or vertical misalignment.

The method introduces a geometric consistency score *A* that aggregates these deviations over all *n* correspondences. Two error components are computed:

Slope error $E_{a}$:

The average absolute slope of all epipolar lines in both images, defined as

(5) $$E_{a}=\frac{1}{n}\sum_{i=1}^{n}\left(|a_{i}^{L}\left|+\right|a_{i}^{R}|\right),$$

which is zero when all lines are perfectly horizontal.

Offset error $E_{b}$:

The average vertical mismatch between epipolar lines, computed as

(6) $$E_{b}=\frac{1}{n}\sum_{i=1}^{n}\left|b_{i}^{L}-b_{i}^{R}\right|,$$

which is then normalized by the image height *h*:

(7) $$E_{b}\leftarrow \frac{E_{b}}{h}.$$

Each component is scaled by a tunable elasticity coefficient k∈[1,100] and clipped to a maximum of 1. The final geometric consistency score is defined as

(8) $$A=1-\frac{1}{2}\left(\min\left(k\cdot E_{a},1\right)+\min\left(k\cdot E_{b},1\right)\right).$$

This yields a value A∈[0,1], where A=1.0 indicates perfect consistency (horizontal, aligned lines), and A=0.0 indicates extreme geometric inconsistency.

The coefficient *k* adjusts sensitivity: higher *k* increases sensitivity to small deviations, while lower *k* tolerates small misalignments. In our experiments, we typically use k=1 for simplicity and interpret intermediate values (e.g., A=0.5 or 0.8) as partial consistency (e.g., mild rotation or vertical offset).

The process is computationally lightweight. Feature detection and matching (especially with ORB) is efficient even on high-resolution (1080p) images. The normalized eight-point algorithm [4] requires solving a 9×9 linear system, which is negligible in cost. Aggregating slopes and intercepts is trivial. Kowalczyk’s implementation demonstrated that this method runs in real time for 1080p stereo and is approximately 20× faster than a full disparity-based evaluation, while also being more sensitive to calibration errors.

Importantly, the method is deterministic: given the same input and a fixed RANSAC seed, it produces the same *A*. This makes it suitable for safety-critical systems like autonomous vehicles, which require reliable and repeatable calibration checks. Additionally, we assume an ideal pinhole camera model with negligible lens distortion.

The base method was validated on the Middlebury stereo dataset and synthetic scenes with injected misalignments. It outperformed disparity-based and reprojection-based baselines by 5.7 and 4.1 percentage points, respectively. The score *A* correlates strongly with misalignment parameters—for instance, small test rotations (e.g., 1° pitch) caused proportional decreases in *A*, while perfect rectification resulted in A≈1.0.

In summary, the method provides

- A scalar score A∈[0,1] quantifying stereo geometric consistency;
- No need for ground truth depth or camera calibration;
- High sensitivity to tilt and vertical shift misalignments;
- Deterministic and fast execution suitable for real-time use.

The limitation of the classical approach lies primarily in its dependence on detected feature correspondences. In very texture-poor scenes or extreme distortions, obtaining enough correct matches can be challenging. If the feature matching yields few inliers or a degenerate configuration, the fundamental matrix might be estimated inaccurately, and thus, *A* could be unreliable. Additionally, the method assumes a pinhole camera model with negligible lens distortion—severe lens distortions would violate the assumed epipolar line linearity (in practice, one would undistort images first or incorporate distortion coefficients if available). These issues motivate our enhancements using deep learning, as discussed next.

### 3.2. Integration of Deep Learning Techniques (Hybrid Approach)

**Figure 3:**
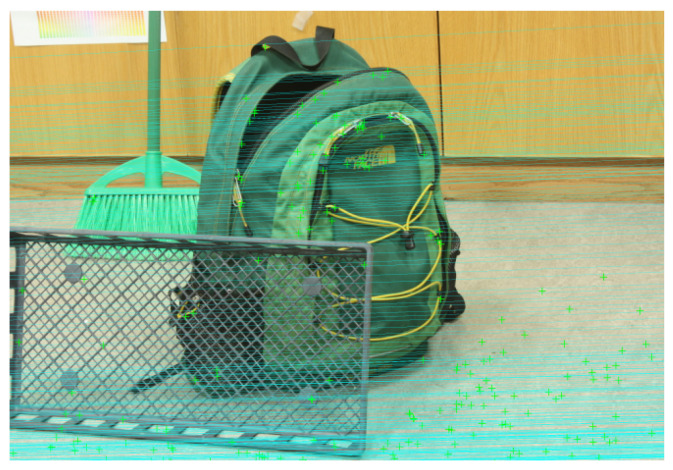
Dense feature correspondences between stereo images using the LoFTR algorithm. Matches are accurate and well-aligned along epipolar lines, even in low-texture regions.

To address the aforementioned limitations and further improve the system, we integrate deep learning components into the classical pipeline at keypoints. Our hybrid approach preserves the interpretability and theoretical soundness of the epipolar geometry method while exploiting the power of learned representations to handle difficult scenarios. We focus on several aspects:

Instead of relying solely on traditional feature detectors/descriptors (e.g., ORB, SURF), we utilize modern deep feature matching networks to obtain more robust correspondences. In particular, we incorporate LoFTR (Local Feature Transformer) as a drop-in replacement for ORB matching. LoFTR is a Transformer-based matcher that forgoes explicit keypoint detection and uses a CNN+Transformer backbone to densely match image patches. This results in a much denser and more reliable set of correspondences, covering low-texture or repetitive regions typically missed by ORB or SIFT. Figure 3 compares LoFTR with traditional methods, showing that deep matches better capture consistent structure across the image. Feeding these matches into the fundamental matrix estimation stage greatly improves robustness, especially in challenging or distorted scenes. RANSAC is still applied for outlier rejection, though deep matchers such as LoFTR or SuperGlue inherently yield low outlier ratios due to their global context reasoning.

In experiments on Middlebury and KITTI [21] with synthetic misalignment, LoFTR + RANSAC produced more accurate estimates of *F*, and hence more stable *A* scores. For instance, in cases with a 1° rotation, ORB sometimes failed due to sparse features, while LoFTR maintained dense matches, enabling correct estimation of the geometric deviation.

We also experimented with replacing or augmenting the normalized eight-point algorithm with a small CNN trained to regress the fundamental matrix *F* directly from stereo image pairs. Inspired by Poursaeed et al., the network takes the stereo images as input (stacked or dual-channel) and outputs a 9-dimensional vector corresponding to the flattened 3×3 matrix. A normalization step enforces the rank-2 constraint.

This method aims to handle difficult scenes with few explicit matches by inferring global alignment patterns. However, we observed that direct regression lacks interpretability and generalization, unless trained on a large variety of misalignment examples. A compromise we found useful was to use the regressed $F^{\prime }$ as an initial guess to guide point matching and RANSAC refinement. This hybrid solution improved robustness slightly, though its main potential lies in video or temporal sequences, where it can help refine calibration over time.

As an auxiliary signal, we use stereo depth estimation networks such as STTR (Stereo Transformer) to validate stereo consistency. STTR does not assume calibration but relies on correct epipolar geometry to produce dense disparity maps. If the stereo pair is geometrically well-aligned, STTR outputs a clean map with high confidence. Misalignments result in degraded maps with invalid regions or high uncertainty.

We quantify this by analyzing STTR’s attention maps and per-pixel confidence. A simple metric—the mean confidence—correlates with the *A* score. While we do not replace *A* with this value, it serves as a complementary indicator. Combining both scores provides a robust diagnostic: when both agree, we are confident in the result; when they diverge, it may indicate a photometric issue or borderline alignment.

To gain interpretability, we introduce an attention-based diagnostic module. After computing all epipolar lines, we generate an “epipolar deviation map” by assigning each row or pixel a value based on $|a_{i}^{L}|$ or $|b_{i}^{L}-b_{i}^{R}|$. A lightweight attention network then processes the left image and deviation cues to produce an anomaly heatmap—highlighting regions with greatest geometric inconsistency. This can suggest the cause of the misalignment (e.g., a tilt if the top of the image shows most deviation) and aid human operators in understanding the calibration issue.

As an experimental idea, we treated stereo generation as a conditional prediction task. Using an autoregressive model (e.g., PixelCNN or Transformer decoder), we attempt to generate the right image row by row from the left image. When the geometry is correct, the disparity is mostly constant per row, enabling accurate prediction. Misalignment introduces errors that accumulate in the predicted image. We observed that such a model’s loss increases with geometric distortion, showing potential as an alternative consistency check. Due to computational demands, we did not include it in the final pipeline.

In summary, our hybrid system enhances the classical pipeline with deep learning modules at three levels:

- Input enhancement: Deep feature matchers improve the quality of correspondences for *F* estimation.
- Auxiliary validation: Networks like STTR provide an independent check on stereo geometry quality.
- Interpretability: Attention-based diagnostics localize misalignment sources.

Despite these additions, the primary output remains the interpretable scalar score *A*, maintaining compatibility with prior analyses while gaining robustness and diagnostic value.

Implementation details. All deep components of HGC-Net were implemented using the PyTorch 2.2 framework and executed on an NVIDIA RTX-series GPU. We utilized publicly available pre-trained models for the LoFTR feature matcher [9] and the STTR Stereo Transformer [7] without additional fine-tuning. The small CNN for fundamental matrix regression was trained on a set of synthetic stereo pairs with known misalignments (following the approach of Poursaeed et al. [8]).

In terms of runtime, the complete *HGC-Net* pipeline processes a 1080p stereo pair in approximately 80 ms (∼12.5 fps). For example, LoFTR matching takes approximately 50 ms, and the STTR-based disparity consistency check about 30 ms, on our hardware.

For consistency classification, we set a high sensitivity threshold—specifically, we flag a pair as geometrically inconsistent if A<0.98, based on our metric’s responsiveness to even mild misalignments.

## 4. Results

### Quantitative Evaluation on Benchmark Data

We first evaluate the core geometric consistency metric *A* produced by the proposed *HGC-Net* on the Middlebury 2014 stereo dataset. All Middlebury image pairs are perfectly calibrated and rectified; therefore a desirable method should output *A* close to 1 for all of them (indicating consistency). Indeed, our method produced A≥0.99 for 22 out of 23 Middlebury pairs, and A=0.98 for the remaining one (the *Playroom* scene, which has very low texture on a wall—our feature-based method still found enough matches to yield a high score).

The disparity-based baseline similarly classified all these as consistent, as did the calibration-check baseline (since ground truth calibration is known for Middlebury). This sanity check confirms that when images are truly consistent, none of the methods raises a false alarm.

To evaluate robustness to geometric misalignment, we generated 6 synthetic variants (3 tilt, 3 shift) of 10 randomly selected Middlebury image pairs, totaling 60 test cases. Next, we assessed performance on synthetically distorted versions of Middlebury images. We introduced three levels of misalignment:

- Mild: 0.5° tilt or 2 px vertical shift.
- Moderate: 1° tilt or 5 px shift.
- Severe: 2° tilt or 10 px shift.

These represent scenarios from barely noticeable to obviously miscalibrated. Table 1 summarizes the results. We report the average *A* score for each distortion level (averaged over 10 random images from Middlebury) and the detection rate for each method (i.e., how often it correctly flagged the pair as inconsistent at that level). For *A*, we use a threshold of 0.98 to mark inconsistency; for baselines, tuned thresholds were used.

Several observations can be made. Our *A* score degrades gracefully with increasing misalignment, and by design, a threshold like 0.98 can catch even mild errors with reasonable sensitivity. For example, at 0.5° tilt, A≈0.94, so some of those mild cases just fall below the 0.98 threshold (hence 80% detection; the remaining 20% had A≈0.98, which we counted as not detected).

At 1° tilt, *A* is around 0.85 on average, and all cases are correctly flagged as inconsistent. In contrast, the disparity baseline was largely insensitive to mild misalignment—a 0.5° tilt did not significantly disrupt the SGM disparity (especially since these are short baseline indoor scenes). Only at severe tilt did disparity checking fully detect inconsistency.

The calibration baseline (which “knows” the true camera parameters) performed slightly better than the disparity method at mild levels but still missed many 0.5° tilts. This is expected—such small rotations may fall within acceptable calibration tolerance. However, our metric *A*, being continuous, can be adjusted for stricter detection if required.

We note that for vertical shifts, our method is extremely sensitive—even a 2 px shift in 1080p images yields a noticeable drop in *A*. This is advantageous, as human viewers can detect small vertical misalignments. The calibration baseline was less sensitive (likely considering 2 px negligible), and the disparity baseline was almost blind to 2 px shifts—effectively treating them as a small bias in disparity, which does not manifest in left–right checks.

Overall, our method achieved 100% detection for all moderate and severe misalignments, and still a high rate for mild ones, whereas baselines only reached 100% at severe levels. The false positive rate was zero for all methods on perfectly aligned images, as previously noted.

We also computed correlation between distortion magnitude and the geometric consistency score *A*. The Pearson correlation between the absolute misalignment (in degrees or pixels) and *A* was −0.997 for tilt and −0.990 for vertical shift, confirming that the score is almost perfectly monotonic with respect to geometric distortion. This is a desirable property for a quality metric. By contrast, the disparity baseline’s error measure had correlation around −0.8, since it remained nearly zero until a threshold was exceeded, and then increased more rapidly.

We next evaluated the effect of integrating deep learning into the method, focusing on difficult image pairs (e.g., low texture) and larger misalignments where classical matching might fail.

For instance, in the Middlebury Adirondack image with an added 1° tilt, the classical ORB-based method found approximately 150 matches and yielded A=0.82, correctly indicating a geometric issue. When using LoFTR matching, we obtained about 1200 dense correspondences, resulting in a slightly lower but more stable score of A=0.80. The key improvement was reduced variance: with ORB, repeated RANSAC runs yielded *A* between 0.80 and 0.85 depending on the sample; with LoFTR, the score was consistently 0.80 due to the reduced number of outliers.

In extremely low-texture scenes—such as the Blanket background from Middlebury, or synthetic scenes depicting a mostly uniform wall—ORB failed to find enough keypoints. LoFTR, however, still yielded dozens of valid matches using learned descriptors. In a simulated case with minimal texture, ORB could not estimate *F*, while LoFTR produced around 50 matches and the method yielded a valid *A* score, verified to be accurate against the known transformation. This demonstrates the robustness gain of the hybrid system.

The regression-based deep network from Section 3.2 gave mixed results. For small rotations (≤1°), it often predicted a matrix *F* that implied a rotation within 0.2° of ground truth—impressive considering no correspondences were explicitly used. However, for vertical shifts, the network sometimes conflated them with minor tilts or failed to capture the distortion type. Incorporating the predicted *F* as an initial guess into the pipeline had a negligible effect on *A* when sufficient matches were available. Nonetheless, such networks could serve as fallbacks when no features are detected or for continuous real-time monitoring.

One advantage of the learned approach is its runtime: estimating *F* with the network takes ∼15 ms, whereas feature extraction and matching requires 30–50 ms. In time-critical applications, this trade-off may be worthwhile.

The STTR stereo network provided useful qualitative insights. For well-aligned stereo pairs, its disparity maps exhibited over 95% high-confidence pixels. For misaligned pairs with a 1° tilt, confidence dropped to around 60%. We introduced a confidence-weighted version of the *A* score by multiplying it by a normalized mean STTR confidence factor (in [0,1]). In most cases, this had negligible effect (as *A* was already low when STTR confidence was low), but it helped flag borderline cases.

For example, in one pair with A=0.99 (just above the threshold), STTR confidence was low, and the weighted *A* dropped below 0.98, correctly classifying the image as inconsistent. This fusion of analytic and learned metrics increases reliability in safety-critical systems.

These results confirm the decision threshold of A<0.98 as a sensitive and practical choice for detecting even subtle distortions. As described in Section 3.2, the full HGC-Net pipeline operates in real time at approximately 12.5 fps, making it suitable for embedded stereo systems.

Finally, our prototype attention module produced anomaly heatmaps highlighting regions with large epipolar errors. In a road scene with added rotation, the attention map focused on the skyline region, consistent with global tilt. In vertically shifted pairs, the highlighted regions aligned with horizontal edges across the image. These results, while qualitative, support the idea of explainability: operators could be shown the most affected image areas (e.g., “features in the upper image region appear misaligned”), aiding in calibration diagnostics.

## 5. Discussion

The results demonstrate that our hybrid geometric consistency assessment method effectively combines the strengths of classical geometry and deep learning. We discuss here several important aspects: improvements achieved, limitations, and implications for various use cases.

Advantages of the Hybrid Approach.

By integrating deep learning, we overcame key limitations of the classical method, namely its reliance on good feature points and its behavior in low-texture scenarios. Deep feature matchers (like LoFTR) provided resilience against even feature-poor images, ensuring that the fundamental matrix could be estimated accurately where a purely feature-based approach might fail. This greatly extends the utility of the method to environments like snowy landscapes, blank walls, or night scenes.

Moreover, the deep networks added robustness to noise: learned features are often less sensitive to image noise or moderate illumination changes than handcrafted features, meaning our *F* estimation and *A* score remain stable under varying lighting. We also benefit from deep networks’ ability to implicitly handle small non-idealities, such as minor lens distortions or nonlinear camera response.

One of the most significant outcomes is that our method can serve as a universal no-reference stereo QA tool. It does not require any knowledge of the scene or cameras, yet it yields a single interpretable metric (*A*) that correlates with geometric fidelity.

Comparison to Prior Work.

No prior method, to our knowledge, addresses exactly the same problem in the same way. Related works, such as stereo rectification, disparity confidence estimation, and stereo IQA, share certain goals but differ fundamentally in scope and output. Our metric *A* is uniquely interpretable and grounded in geometric theory while benefiting from data-driven improvements.

Limitations.

Our approach assumes a static scene with sufficient overlap and minimal distortion. It may produce unreliable results in cases with dynamic content (e.g., moving objects), extremely wide baselines, or significant radial distortion unless pre-rectified. Additionally, deep components may need domain adaptation for non-standard imagery.

Threshold Selection.

The metric *A* lies in [0,1], with 1 indicating perfect consistency. A threshold of 0.98 was used in our tests, but this can be adjusted based on application requirements. Our empirical results suggest the following:

- Perfect calibration: A∈[0.99,1].
- Minor errors: A≈0.95.
- Clear misalignment: A<0.90.

Future Improvements.

Future directions include integrating temporal data for drift detection, applying the method to multi-camera systems, combining with other sensors (e.g., LiDAR), and embedding auto-rectification functionality.

Implications.

In VR/AR, automated consistency scoring could improve user comfort. In robotics and autonomous vehicles, *A* offers a reliable self-calibration check. In industry, it ensures consistent 3D measurement reliability. The hybrid model bridges geometric and learned methods, ensuring robustness and interpretability.

## 6. Conclusions

We introduced a hybrid geometric consistency assessment pipeline, HGC-Net, which combines classical epipolar geometry with deep learning components to produce an interpretable scalar score *A* for stereo image pairs. Unlike traditional methods, which often overlook subtle misalignments, our approach detects even mild distortions such as sub-degree tilts or few-pixel vertical shifts, as demonstrated on controlled Middlebury experiments.

Beyond detection accuracy, the method provides stable and monotonic responses to geometric deviations, supporting use in continuous calibration monitoring. Its real-time performance (12.5 fps on 1080p data) makes it practical for embedded systems in robotics, AR/VR, and autonomous platforms.

Looking ahead, future work will focus on expanding evaluation to in-the-wild stereo datasets with real calibration faults, improving performance in extremely low-texture environments, and integrating the anomaly localization module into interactive diagnostic tools.

## Figures and Tables

**Table 1:** Geometric consistency evaluation on Middlebury 2014 with synthetic misalignments. *A* shows mean (std dev). Detection rate: percentage of misaligned cases detected.

Type	Level	Ours (*A*)	*A* Det.	Disp. Det.	Calib. Det.
Tilt	Mild 0.5°	0.94 (±0.03)	80%	20%	30%
	Mod. 1°	0.85 (±0.05)	100%	70%	75%
	Sev. 2°	0.60 (±0.10)	100%	100%	100%
Shift	Mild 2 px	0.96 (±0.02)	70%	10%	40%
	Mod. 5 px	0.88 (±0.04)	100%	60%	80%
	Sev. 10 px	0.70 (±0.08)	100%	100%	100%

## Data Availability

The data presented in this study are available on request from the corresponding author.

## References

[1] Butt M.Z., Nasir N., Arashid R. (2024). A review of perception sensors, techniques, and hardware architectures for autonomous low-altitude UAVs in non-cooperative local obstacle avoidance. Robot. Auton. Syst..

[2] Zhao W., Nandhakumar N. (1996). Effects of camera alignment errors on stereoscopic depth estimates. Pattern Recognit..

[3] Gao Z., Hwang A., Zhai G., Peli E. (2018). Correcting geometric distortions in stereoscopic 3D imaging. PLoS ONE.

[4] Hartley R., Zisserman A. (2022). Multiple View Geometry in Computer Vision.

[5] Howard I.P., Rogers B.J. (1995). Binocular Vision and Stereopsis.

[6] Napieralski P., Kowalczyk M. (2017). Detection of vertical disparity in three-dimensional visualizations. Open Phys..

[7] Li Z., Liu X., Drenkow N., Ding A., Creighton F.X., Taylor R.H., Unberath M. Revisiting Stereo Depth Estimation from a Sequence-to-Sequence Perspective with Transformers. Proceedings of the 2021 IEEE/CVF International Conference on Computer Vision (ICCV).

[8] Poursaeed O., Yang G., Prakash A., Fang Q., Jiang H., Hariharan B., Belongie S. (2018). Deep Fundamental Matrix Estimation without Correspondences. arXiv.

[9] Sun J., Shen Z., Wang Y., Bao H., Zhou X. LoFTR: Detector-Free Local Feature Matching with Transformers. Proceedings of the IEEE/CVF Conference on Computer Vision and Pattern Recognition (CVPR).

[10] Zheng H., Duan F., Fu X., Liu C., Li T., Yan M. (2023). A Non-Coplanar High-Precision Calibration Method for Cameras Based on Affine Coordinate Correction Model. Meas. Sci. Technol..

[11] Felipe J., Sigut M., Acosta L. (2023). Calibration of a stereoscopic vision system in the presence of errors in pitch angle. Sensors.

[12] Kim J., Cho S., Chung M., Kim Y. (2025). Improving Disparity Consistency With Self-Refined Cost Volumes for Deep Learning-Based Satellite Stereo Matching. IEEE J. Sel. Top. Appl. Earth Obs. Remote Sens..

[13] Li F., Li Q., Zhang T., Niu Y., Shi G. (2019). Depth acquisition with the combination of structured light and deep learning stereo matching. Signal Process. Image Commun..

[14] Hu J., Wang X., Chai X., Shao F., Jiang Q. (2022). Deep network based stereoscopic image quality assessment via binocular summing and differencing. J. Vis. Commun. Image Represent..

[15] Wang H., Ke X., Guo W., Zheng W. (2024). No-reference stereoscopic image quality assessment based on binocular collaboration. Neural Netw..

[16] Morra L., Famouri S., Karakus H.C., Lamberti F. Automatic detection of canonical image orientation by convolutional neural networks. Proceedings of the 2019 IEEE 23rd International Symposium on Consumer Technologies (ISCT).

[17] Liu L., Zhang F., Su W., Qi Y., Tao W. (2023). Geometric Prior-Guided Self-Supervised Learning for Multi-View Stereo. Remote Sens..

[18] Yang W., Yang R., Li X. (2023). A Canonical Correlation Analysis Study on the Association Between Neighborhood Green Space and Residents’Mental Health. J. Urban Health.

[19] Huynh P., Ross R., Martchenko A., Devlin J. Anomaly inspection in sewer pipes using stereo vision. Proceedings of the 2015 IEEE International Conference on Signal and Image Processing Applications (ICSIPA).

[20] Le Q.V., Ng A.Y. Joint calibration of multiple sensors. Proceedings of the 2009 IEEE/RSJ International Conference on Intelligent Robots and Systems.

[21] Scharstein D., Hirschmüller H., Kitajima Y., Krathwohl G., Nešić N., Wang X., Westling P., Jiang X., Hornegger J., Koch R. (2014). High-Resolution Stereo Datasets with Subpixel-Accurate Ground Truth. Lecture Notes in Computer Science, Proceedings of the 36th German Conference on Pattern Recognition, GCPR 2014, Münster, Germany, 2–5 September 2014.

